# *Polo*: an open-source graphical user interface for crystallization screening

**DOI:** 10.1107/S1600576721000108

**Published:** 2021-02-19

**Authors:** Ethan T. Holleman, Erica Duguid, Lisa J. Keefe, Sarah E. J. Bowman

**Affiliations:** a Hauptman-Woodward Medical Research Institute, 700 Ellicott Street, Buffalo, NY 14203, USA; b Industrial Macromolecular Crystallography Association Collaborative Access Team, Advanced Photon Source, Argonne National Laboratory, 9700 South Cass Avenue, Lemont, IL 60439, USA; c Department of Biochemistry, Jacobs School of Medicine and Biomedical Sciences at the University at Buffalo, Buffalo, NY 14023, USA

**Keywords:** crystallization, crystal imaging, machine learning, open-source graphical user interfaces

## Abstract

*Polo* is a Python-based graphical user interface designed to streamline viewing and analysis of images to monitor crystal growth, with a specific target to enable users of the High-Throughput Crystallization Screening Center at Hauptman-Woodward Medical Research Institute (HWI) to efficiently inspect their crystallization experiments. *Polo* aims to increase efficiency, reducing time spent manually reviewing crystallization images, and to improve the potential of identifying positive crystallization conditions. *Polo* provides a streamlined one-click graphical interface for the Machine Recognition of Crystallization Outcomes (MARCO) convolutional neural network for automated image classification, as well as powerful tools to view and score crystallization images, to compare crystallization conditions, and to facilitate collaborative review of crystallization screening results. Crystallization images need not have been captured at HWI to utilize *Polo*’s basic functionality. *Polo* is free to use and modify for both academic and commercial use under the terms of the copyleft GNU General Public License v3.0.

## Abstract 2

A multi-platform open-source Python-based graphical user interface has been developed to provide access to automated classification and data management tools for biomolecular crystallization screening.

## 1. Introduction   

Growing high-quality protein crystals is a major bottleneck in crystal-based structure determination, which accounts for ∼90% of all structures in the Protein Data Bank (PDB) (Derewenda, 2011 ▸; Fazio *et al.*, 2014 ▸; Lynch *et al.*, 2020 ▸). This bottleneck is a result of both the inherent difficulty of crystallizing macromolecular samples and the labor-intensive process of traditional experimental protocols which depend on the manual preparation and examination of dozens to hundreds of crystallization conditions (McPherson & Gavira, 2014 ▸). Both challenges have made high-throughput crystallization screening experiments an appealing alternative. High-throughput (HT) crystallization facilities, such as the Crystallization Center at Hauptman-Woodward Medical Research Institute (HWI), utilize liquid-handling robotics and state-of-the-art imagers to investigate crystallization of macromolecular samples in hundreds of non-redundant chemical conditions (also called cocktails or screening conditions) (Luft *et al.*, 2003 ▸). At the Crystallization Center, experiments set up in 1536 wells in a single HT microassay plate provide efficient screening and exploration of a large non-redundant chemical parameter space to identify positive crystallization outcomes.

HT crystallization screening typically takes place at centralized facilities. At the Crystallization Center at HWI, researchers ship their macromolecular samples to the facility where experimental setup is handled on-site. During the experiment, each of the 1536 wells is imaged multiple times to probe which conditions promote crystal growth; the resulting images are made available for remote review. HT screening reduces the time researchers need to devote to crystallization experiments and greatly increases the variety of screening conditions tested. However, this creates a challenge, as even in HT experiments most chemical conditions will still fail to produce macromolecular crystals. This results in a needle-in-the-haystack problem requiring researchers to carefully and manually review potentially tens of thousands of images to identify successful crystallization conditions (Luft *et al.*, 2011 ▸). At the Crystallization Center at HWI, each 1536 screening experiment generates a minimum of 13 824 images over the six-week time window. The sheer number of images in HT screening experiments has made automated image classification algorithms highly sought after, with numerous machine-learning approaches applied (Liu *et al.*, 2008 ▸; Sigdel *et al.*, 2013 ▸; Hung *et al.*, 2014 ▸). A major step forward in tackling this problem came in 2008 with the establishment of a repository of classified HT Crystallization Center images (Snell, Lauricella *et al.*, 2008 ▸; Snell, Luft *et al.*, 2008 ▸), which opened the door to researchers looking to train deep convolution neural networks. Later, Yann & Tang (2016 ▸) reported a *CrystalNet* model. More recently, Bruno *et al.* (2018 ▸), in a collaboration between a number of crystallization facilities from around the world, pharmaceutical companies, universities and GoogleBrain, reported the Machine Recognition of Crystallization Outcomes (MARCO) model. MARCO is a convolutional neural network trained on a repository of over 490 000 labeled images from five institutions (Bruno *et al.*, 2018 ▸). The MARCO algorithm results in a success rate of over 90% in classifying test set images to their correct classes for four classifications (Crystals, Precipitate, Clear or Other). The MARCO model is open source, requiring *TensorFlow* or *TensorFlow Lite* (Vanhoucke, 2018 ▸), but implementation of the algorithm in many crystal screening laboratories is challenging, which has limited the practical use of the MARCO model. MARCO has been implemented in commercial-level closed-source programs (notably the Formulatrix *ROCK MAKER* software suite), and Merck has generated a web interface for accessing *ROCK MAKER* images with the potential to implement MARCO (Lesburg, 2020 ▸). The Collaborative Crystallization Centre (C3) protein crystallization facility in Parkville, Australia, has incorporated MARCO into the viewing, scoring and optimization tool ‘*See3*’ available to users of the C3 facility (Rosa *et al.*, 2020 ▸). However, to the best of the authors’ knowledge, there has been no graphical user interface (GUI) program targeted to individual researchers that incorporates the MARCO model. Our GUI, *Polo*, seeks to fill this gap by providing one-click use of the MARCO model with intuitive image viewing and classification interfaces. In addition to its easy-to-use visual interfaces, the capabilities of *Polo* enable access to metadata such as cocktail components, as well as viewing multiple image modalities. *Polo* complements MARCO by abstracting data management away from direct user control while maintaining accessibility by providing tools to export data to easily parsable formats such as csv and JSON. *Polo* also facilitates automatic generation of more human-readable files, such as *PowerPoint* presentations. Further, *Polo* is free to use and modify for both academic and commercial use under the terms of the permissive GPL v3.0 software license.

## 2. Hardware and software requirements   

*Polo* has been tested on Windows 10, Mac operating system versions High Sierra, Mojave and Catalina, and Ubuntu versions 18.04 (Bionic Beaver) and 20.04 (Focal Fossa). The minimum specifications of any test machine were two processor cores and 4 GB of RAM. Note that use of the MARCO model is CPU intensive and faster processors will result in significantly decreased classification times. It is recommended to use a display with a resolution of at least 1920 × 1080 to allow easy viewing of all interfaces. The Mac and Windows versions of *Polo* do not require the installation of additional software or programming languages, but Ubuntu users will need to install the *unrar* program to interact with rar archive files. Instructions to do so are available in the *Polo* manual and on the *Polo* website.

## 3. Graphical interfaces   

Allowing for easy use of the MARCO model alone, while improving accessibility of the algorithm, would not necessarily prove beneficial or increase adoption by average users without an interface that effectively conveys classification results, contextualizes them in the presence of all available imaging data, and allows for easy manual classification and review of model results. For this reason, *Polo* includes several interfaces for reviewing and classifying crystallization images, with or without use of the MARCO model.

### 3.1. Image organization and terminology   

Image organization and terminology within the *Polo* interface have been based on protocols at the Crystallization Center at HWI, and an understanding of the experimental protocols and terms that have motivated *Polo*’s design is necessary for developing an understanding of the interfaces. Typically, when a researcher sends a macromolecular sample for screening, it will be robotically dispensed onto a screening plate that is composed of hundreds of screening wells, each containing a unique chemical cocktail. As the experiment progresses in time, each well will be imaged, creating collections of images called runs; each represents the state of the experiment at a specific point in time. Runs are represented digitally as directories of images and may optionally contain metadata files describing the experimental conditions. Images are captured with standard bright-field (visible) microscopy. In addition, the Crystallization Center uses a Formulatrix Rock Imager 1000 with SONICC (second-order nonlinear imaging of chiral crystals), which enables ultraviolet two-photon excited fluorescence (UV-TPEF) and second-harmonic generation (SHG) microscopies. UV-TPEF and SHG imaging modalities can be used to differentiate macromolecular crystals from precipitate and salt crystals and to identify sub­micrometre-sized crystals (Haupert & Simpson, 2011 ▸; Madden *et al.*, 2011 ▸). Positive SHG images are high contrast with chiral crystals appearing white against a black background. One limitation of SHG imaging for crystal detection is that crystals with higher symmetry will generate weak or nondetectable SHG signals. Nevertheless, it has been estimated that approximately 84% of protein crystals are expected to have positive SHG signals (Haupert *et al.*, 2012 ▸). UV-TPEF is a complementary technique in which concentrated protein samples with aromatic residues will fluoresce, resulting in white spots. Inspecting images from visible, UV-TPEF and SHG modalities allows for rapid identification of conditions in which proteins will crystallize. Additionally, using UV-TPEF and SHG modalities can enable identification of crystals that may otherwise be classified as precipitate or clear. The MARCO algorithm has been trained only on bright-field images but using the combination of all three images when examining MARCO classifications should enable better human classification of the crystallization screening experiment.

In *Polo*, each of the supplementary imaging modalities available from the Crystallization Center HT screening is referred to as an alternative spectrum. *Polo* reflects this experimental structure by organizing individual runs first by their respective sample, then by date of imaging and finally by microscopy method. Additionally, to distinguish between classifications made by the user and those calculated by the MARCO model, these classifications are referred to as human and MARCO, respectively. For simplicity and ease of integration with MARCO, image classifications are made from one of four possible categories: Crystals, Precipitate, Clear or Other.

### 3.2. Data import interfaces   

*Polo* provides users with interfaces for importing their screening runs that have already been downloaded from the Crystallization Center (import from local machine) or by using remote file transfer protocol (FTP). Local files can be imported using either a file browser or drag-and-drop interfaces. For accessing and downloading image data from FTP servers, *Polo* provides a built-in FTP client built on Python’s *ftplib* package (Fig. S1 in the supporting information). The FTP browser can be used to connect to any FTP server available over the internet and is not limited to connections served by HWI.

After a run is imported, whether using FTP or a local file, it will be displayed in the Samples window which organizes runs by sample and imaging date. Runs whose sample cannot be determined will be imported under the name ‘Non-HWI Run’. Selecting a run from the list in the Samples window by double clicking will open it in all other interfaces. Individual runs within the sample can be classified with the MARCO model by selecting the run and then the Classify Selected Run button, or all runs within the sample can be classified by left-clicking the sample and selecting Classify All Runs.

### 3.3. Slideshow Viewer   

**Figure 1:**
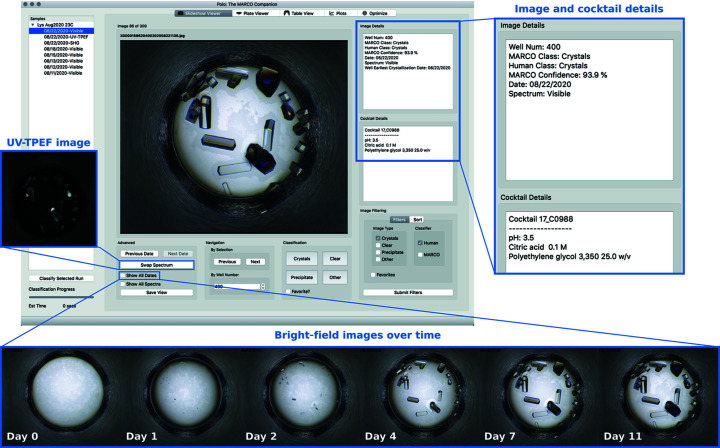
Screenshot of the Slideshow Viewer interface displaying bright-field image of Well Num 400 of a lysozyme sample set up at the Crystallization Center. Crystals are clearly visible in this well, which contains Cocktail C0988. Image Details and Cocktail Details are shown in the inset to the right. Researchers can navigate between images within the currently selected run using the Next and Previous image buttons or directly to a specific well number by typing in the well number associated with the desired image into the By Well Number box. Images can be classified via mouse clicks or keyboard shortcuts using the buttons provided under the Classification section (Crystals = 1, Clear = 2, Precipitate = 3, Other = 4). Classifying an image will automatically advance the Slideshow Viewer to the next image. Particularly interesting images can be chosen as Favorites and selected later using Image Filtering. Using the Swap Spectrum button will show UV-TPEF and SHG imaging modalities if available (UV-TPEF shown in inset to the left). Show All Dates will generate a time course of images of this particular well (shown at the bottom). Any individual image displayed by the Slideshow Viewer can also be exported as a png file using the Save View button.

*Polo*’s Slideshow Viewer enables viewing of experimental images whether they have been classified by the MARCO model or not. Image data are integrated with associated metadata, including human classifications, MARCO classifications and the associated confidence level, the well number, the modality used to capture the image, and the cocktail details that constitute the screening condition. Image metadata and cocktail information are displayed in the Image Details and Cocktail Details text browsers, respectively (Fig. 1 ▸). The Slideshow Viewer also facilitates filtering images shown by using either human or MARCO classification. The order of the images can be adjusted by sorting the filtered results, which allows images to be ordered by well number, cocktail number or MARCO confidence. Anecdotally, we have observed selection of MARCO crystal class images and sorting by MARCO confidence to be particularly effective at quickly identifying promising screening conditions within a run.

If a researcher has imported multiple runs of the same sample, the Slideshow Viewer can take advantage of all additional imaging data via multi-image views. If multiple time points of imaging are available, the Slideshow Viewer can display timeline views that illustrate the progression of each well (Fig. 1 ▸). Researchers also can navigate through their images in time with the Previous Date and Next Date buttons, which allow for navigation through the complete imaging history of a particular screening well. This provides another method for viewing potential crystal growth information through the progression of the experiment. If alternative imaging modalities are available for the selected run, such as UV-TPEF and SHG, these can be viewed alongside the bright-field images of the wells to aid in confirming or, just as importantly, in ruling out, the presence of macromolecular crystals in an image.

### 3.4. Plate Viewer   

**Figure 2:**
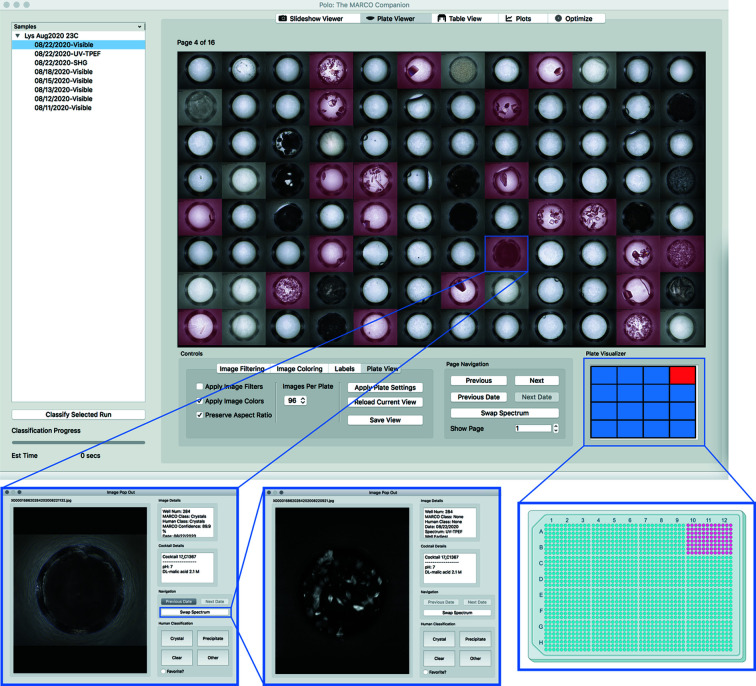
Screenshot of the Plate Viewer interface. (Top) 96 of the 1536 wells in the microassay plate with MARCO classified wells for this subset colored in red. (Bottom) Left: Image Pop Out for one of the wells with crystals in this subset (hidden by precipitate but correctly classified by MARCO). Middle: the Swap Spectrum button changes the image to UV-TPEF, in which the crystals are more clearly apparent. Right: schematic of how the Plate Visualizer indicates where on the 1536 microassay plate the images are from. The 96 images shown are from the upper right 1/16th of the 32 × 48 grid microassay plate.

It is sometimes desirable to review multiple images in an array and scan through those images in search of successful crystallization conditions. *Polo* accommodates this technique through its Plate Viewer interface, which allows viewing grids of 16, 64 or 96 images that represent subsections of the entire 1536 microassay screening plate (Fig. 2 ▸). The Plate Visualizer window displays a representation of what portion of the 1536 HT microassay plate the user is currently viewing. Images can be colored by their MARCO or human classification to facilitate easy identification of image classes, similar to the functionality in the Slideshow Viewer filtering and sorting functions. The interface provides a variety of colors, as well as the option to shade images that do not meet the researcher’s criteria. All image adjustments are made in memory and do not affect stored images. Just like the Slideshow Viewer interface, if time-resolved or alternative spectra imaging runs are available, they may be shown for an entire grid of images. Images displayed by the Plate Viewer can also be exported to png files. Exported images will include any coloring, opacity adjustments or labels applied by the Plate Viewer. Once an ‘interesting image’ has been identified, it can be viewed in greater detail by clicking on it, which will open the selected image in an Image Pop Out view (Fig. 2 ▸). This pop-out viewer is based on the Slideshow Viewer interface and provides equivalent functionality, but for a single image.

### 3.5. Additional interfaces   

*Polo* also includes three other interfaces to supplement the main Slideshow Viewer and Plate Viewer interfaces.

#### 3.5.1. Table View   

**Figure 3:**
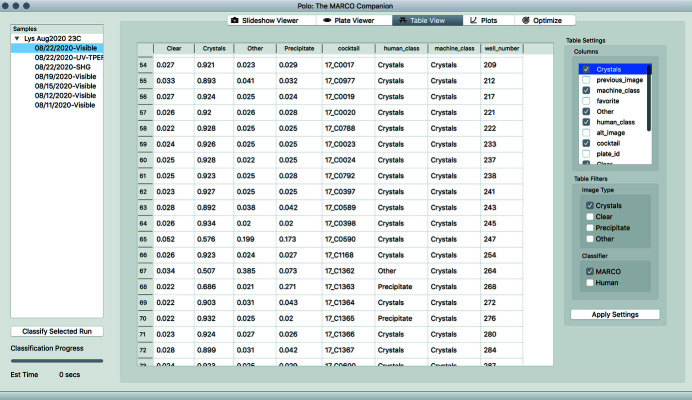
Screenshot of Table View displaying data for all MARCO classified images of a lysozyme sample imaged on 8/22/2020. Similar to the Slideshow Viewer interface, data displayed can be filtered by either MARCO or human classification using the checkboxes located in the lower right under the Table Filters box. Additionally, the type of data that are displayed in the columns can be controlled by using the checkboxes in the upper right of the interface under the Table Settings box.

A non-visual presentation of screening data, the Table View presents run data in a tabular format (Fig. 3 ▸). The Table View will show a variety of metadata once images have been classified by MARCO or by a human scorer (or both). It includes filtering functions equivalent to those found in the Slideshow Viewer and the Plate Viewer. Data displayed can be controlled in this interface by checking or unchecking column headers.

#### 3.5.2. Plots   

*Polo* provides a few basic plots using the *matplotlib* (https://matplotlib.org/) Python package. Plots are accessible under the Plot tab and can be easily modified, resized or exported to png image files using the controls available in the Plot interface. The Plot interface includes ways to visualize human classification progress for the experiment, to compare human classifications against MARCO classifications and to assess the overall accuracy of the MARCO model against human classifications.

#### 3.5.3. Optimize   

Once successful crystallization conditions are identified, optimization is often required to produce diffraction-quality crystals. Optimization often occurs by varying concentrations of macromolecule and cocktail components in 24- to 96-well crystallization plates, where a precipitant is varied along each axis of the plate creating a gradient of conditions around the original positive hit condition. Manual calculations to determine the correct dilutions of precipitants for each well can be time consuming and error prone. *Polo*’s Optimize interface provides a screen builder tool that enables users to generate these optimization screens automatically and export the results to HTML files for reference while in the laboratory. Optimization screens use the successful crystallization cocktail as the midpoint for generating the chemical gradients. Along with their screening-plate dimensions, the researcher enters the screening-plate well volume, the stock concentration of each reagent and the variance in concentration along each step in the gradient as a percentage. The Optimize interface then calculates the required dilutions for each well in the screening plate.

### 3.6. Export   

*Polo* provides the option to export image data, metadata and classifications to several file formats for use by both *Polo* and non-*Polo* users. These tools will be especially helpful in further analyses of crystallization metadata.

#### 3.6.1. *Polo*-specific file formats   

A common issue for crystallization screening is maintaining the association of images, metadata, chemical conditions and classifications in a way that can be easily shared with others. To alleviate this time-consuming issue, *Polo* facilitates saving runs in the *xtal* file format. The *xtal* format is JSON-based and encodes image classifications, chemical conditions and image data in the same file. At the cost of large file size, *xtal* files enable high portability as images, classifications and chemical data are contained in a single file. These files can be shared amongst collaborators who are also using *Polo*. In addition, *Polo* enables export of chemical and classification data to mso files, which do not include encoded images and provide a lighter-weight solution for sharing image classifications and cocktail formulations. Detailed documentation on both file formats can be found at the *Polo* website (see Section 5).

#### 3.6.2. *Polo* agnostic file formats   

For researchers who wish to export their classifications to more widely used file formats, *Polo* provides several options. Researchers can choose to export chemical and classification data to csv files readable by standard spreadsheet programs like Microsoft *Excel* or OpenOffice *Calc*, or to JSON files for which most modern programming languages have built-in packages for parsing. We hope the variety of text-based file formats available will aid researchers seeking a repeatable and standardized method for recording their image classifications and successful screening conditions.

**Figure 4:**
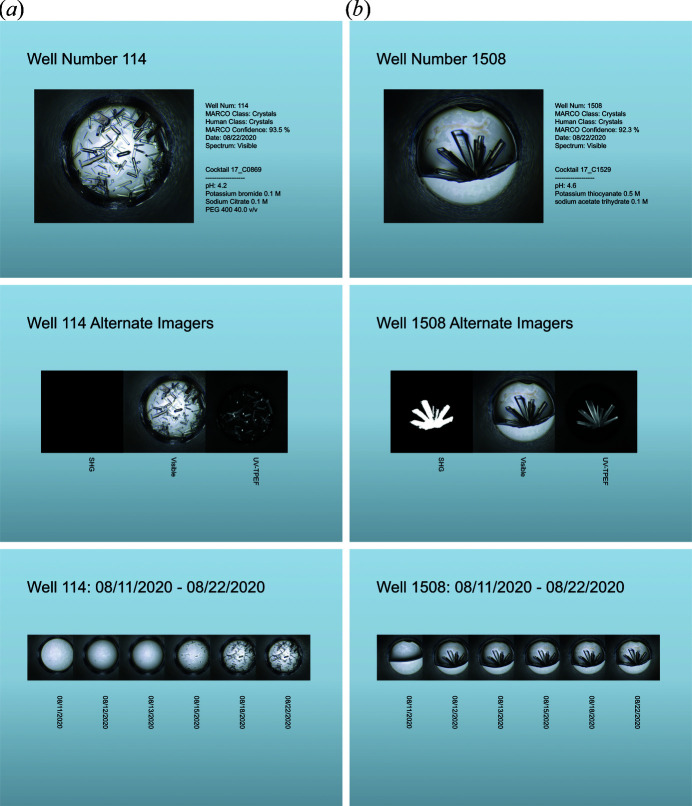
*PowerPoint* slides generated using the Presentation Exporter interface. (*a*) Well 144 (Cocktail C0869) and (*b*) well 1508 (Cocktail C1529) of an HT 1536 experimental setup of lysozyme. Up to three slides are generated per well. (Top) Detail slide with bright-field image and all metadata. (Middle) Alternative image slide which shows the visible image alongside alterative spectra. Lysozyme crystallizes in a number of conditions; many of these crystal growth conditions yield very weak SHG signals that are typically not detected in the HT screening. Well 144 has no SHG signal but clear crystal growth with the UV-TPEF signal verifying protein-containing crystals. Well 1508 reveals positive lysozyme crystallization using all three modalities. (Bottom) A timeline slide which shows the bright-field image from the export run with all available visible images of the selected well.

While text-based representations of screening results are helpful for data management and machine parsing, sharing results with collaborators often necessitates more visual presentation. For this reason, *Polo* incorporates an interface for automatically creating presentation files, the Presentation Exporter (Fig. S2). This eliminates the need to manually gather screening images and their metadata, empowering users to select screening images based on classification and to generate presentations that incorporate all images and associated metadata available in the Slideshow Viewer interface, including time-resolved and multi-spectra views (Fig. 4 ▸). This reduces the time researchers must spend assembling presentation materials, making it easier for users to share results with increased frequency and accuracy. Programmatically generating presentation materials has the added benefit of enforcing a degree of standardization to the presentation of crystallization results, increasing both readability and machine parsability.

## 4. Input requirements and current limitations   

*Polo* can currently accept folders, or rar archives of images of standard file formats including jpeg, png and gif. Once imported, at minimum, images can be classified using the MARCO model and reviewed using the Slideshow Viewer and the Plate Viewer. Classifications can be exported to any included file format. *Polo* was written primarily for the Crystallization Center user base and is based on the Center’s experimental protocols and data management schemas (Fig. S3). This currently limits some of *Polo*’s functionality to non-HWI researchers as it is not feasible to exhaustively anticipate all file formats, naming protocols, data organization schemes *etc*. that may or may not be utilized by other crystallization screening experiments. Users with some experience in Python object-oriented programming should find the *Polo* source code flexible enough to create the necessary functionality required by their experimental and data management protocols (Fig. S4).

## 5. Accessibility and availability   

*Polo* is written in Python and uses the *PyQt* package from Riverside Computing as the graphical engine. All software included or used in *Polo*’s implementation is open source and, at minimum, is free to use or modify provided that any derivative work also remains free and open source. A complete list of dependencies is available in Table S1 or on the *Polo* GitHub page (https://github.com/Hauptman-Woodward/Marco_Polo). Modification, improvement or customization of *Polo*’s source code is encouraged. A self-installing distribution is available for Windows 10 and binary executables are available for Mac OS versions greater than or equal to High Sierra and Ubuntu Linux greater than or equal to 18.04. Current and previous releases of *Polo* can be downloaded from the GitHub releases page (https://github.com/Hauptman-Woodward/Marco_Polo/releases). Currently, root access is required to mark the Mac and Ubuntu distributions as executable. The Windows 10 distribution can be run without administrator privileges. In-depth installation instructions, video tutorials, sample data and source code documentation are available at the *Polo* website (https://hauptman-woodward.github.io/Marco_Polo/).

## 6. Outlook   

*Polo* is an ongoing project under active development. In the future we plan to involve software developers and crystallographers from other organizations and crystal screening facilities to create a more universal application for structural biologists. We also hope to begin integrating tools for more in-depth chemical analysis of successful screening conditions. As the MARCO algorithm has been trained using bright-field images, the classifying function in *Polo* is only designed to perform using this type of image. We note the need to extend the classification capabilities to also include information from alternative imaging modalities, such as UV-TPEF and SHG. *Polo* in the current version provides the capability to view alternative imaging methods alongside the classified images, and we hope to extend these capabilities in the future. Potential contributors are encouraged to contact the authors, to suggest source code modifications and extensions, and to draft pull requests on *Polo*’s GitHub site to aid in the further development of these tools.

## Supplementary Material

Table S1: Dependencies required. FIgs. S1 to S4. DOI: 10.1107/S1600576721000108/ei5066sup1.pdf
