# Supplementary material for: Polo: an open-source graphical user interface for crystallization screening
Source: J Appl Crystallogr. 2021 Feb 19;54(Pt 2):673–9. doi: 10.1107/S1600576721000108 (PMC8056757; doi:10.1107/S1600576721000108)
Supplement: Supplementary file 1 [file j-54-00673-sup1.pdf]

JOURNAL OF
APPLIED
CRYSTALLOGRAPHY

Volume 54 (2021)

Supporting information for article:

Polo: An Open-Source Graphical User Interface for Crystallization Screening

Ethan T. Holleman, Erica Duguid, Lisa J. Keefe and Sarah E. J. Bowman

Package	Version
absl-py	0.9.0
altgraph	0.17
astor	0.8.1
certifi	2018.8.24
chardet	3.0.4
cycler	0.10.0
gast	0.3.3
google-pasta	0.2.0
grpcio	1.30.0
h5py	2.10.0
idna	2.1
importlib-metadata	1.7.0
Jinja2	2.11.2
Keras-Applications	1.0.8
Keras-Preprocessing	1.1.2
kiwisolver	1.1.0

lxml	4.5.2
macholib	1.14
Markdown	3.2.2
MarkupSafe	1.1.1
matplotlib	3.0.3
molmass	2019.1.1
numpy	1.18.5
Pillow	7.2.0
protobuf	3.12.2
PyInstaller	3.6
pyparsing	2.4.7
PyQt5	5.15.0
PyQt5-sip	12.8.0
python-dateutil	2.8.1
python-pptx	0.6.18
requests	2.24.0
six	1.15.0
tensorboard	1.14.0
tensorflow	1.14.0

tensorflow-estimator	1.14.0
termcolor	1.1.0
urllib3	1.25.10
Werkzeug	1.0.1
wrapt	1.12.1
XlsxWriter	1.2.9
zipp	1.2.0

Table S1 Complete list of all Polo dependencies and versions at the time of publication.

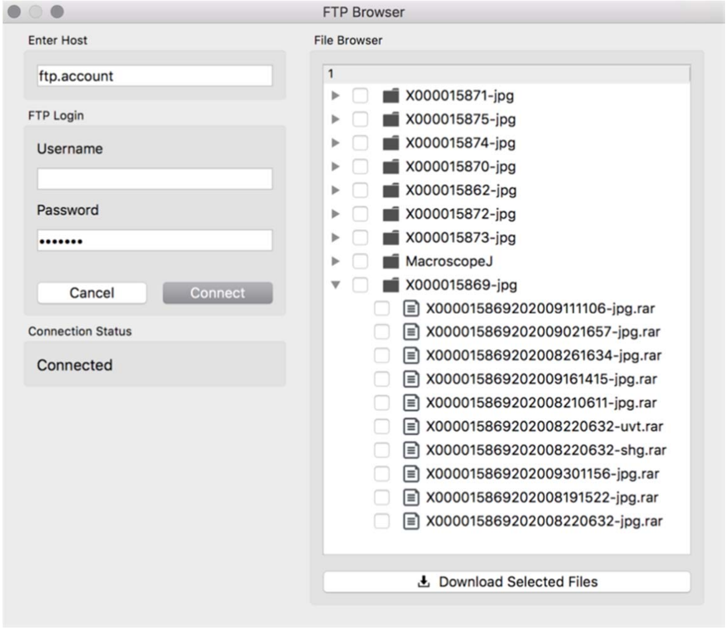

Figure S1 Screenshot of the FTP connected to the HWI server and displaying the files and directories that are available to the user for download. Files can be downloaded individually or by selecting an entire directory. Once a download is initialized by selecting the *Download Selected Files* button the FTP browser closes and downloading begins in the background, allowing the user to continue interacting with other interfaces.

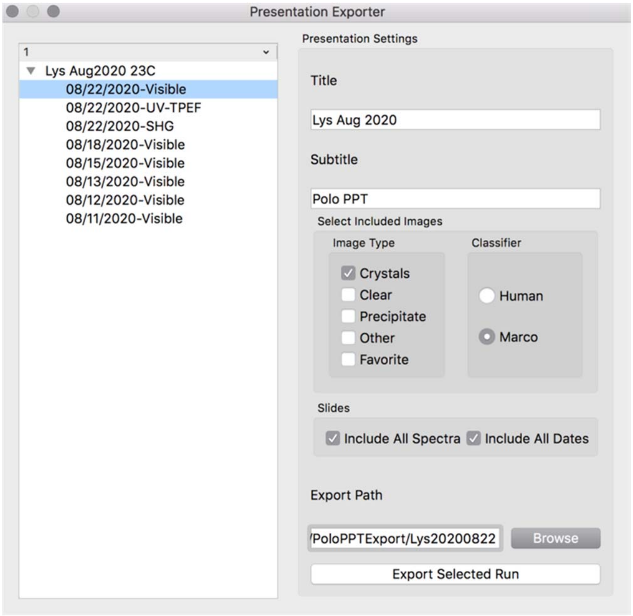

Figure S2 Polo's *Presentation Exporter* interface allows users to export a selection of images. A presentation file is exported that can be viewed in standard presentation programs such as PowerPoint of OpenOffice Impress. Users select the specific classification for a given imaging run date, with additional functionality to *Include All Spectra* and *Include All Dates*.

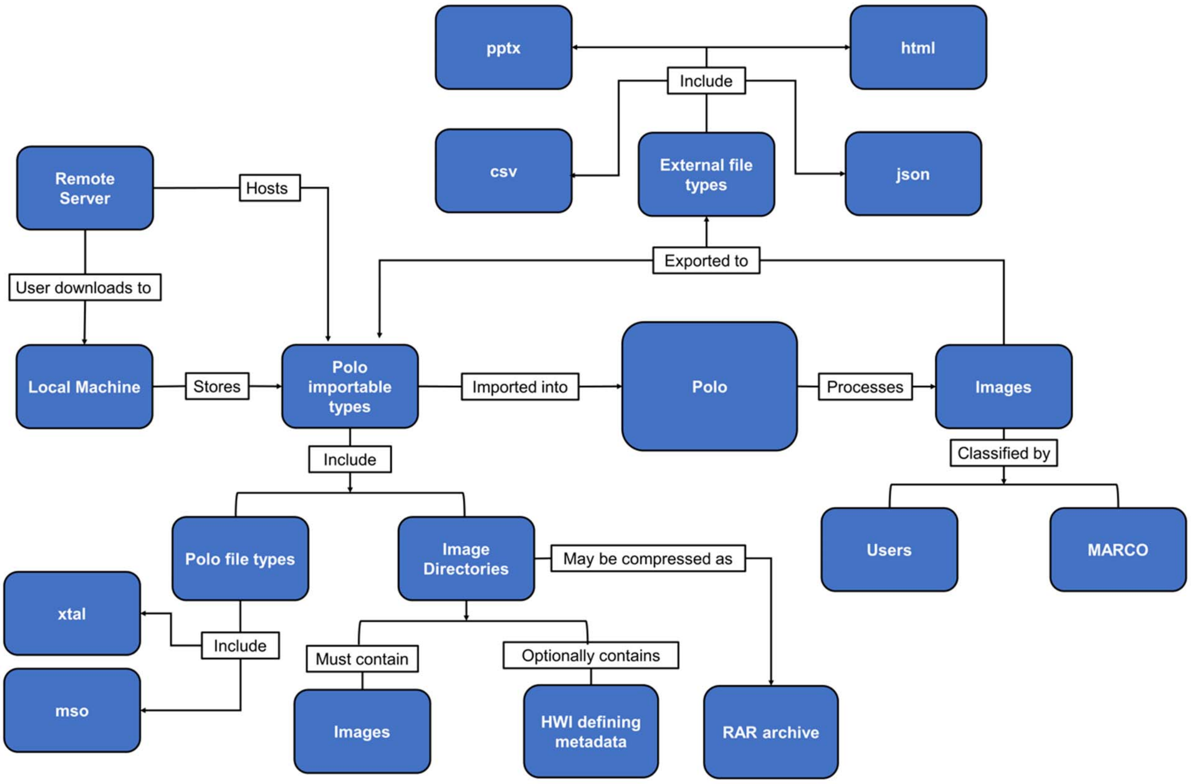

Figure S3 Flowchart of general software operation and data schema from the user perspective. Generally, a user will download screening images from a remote server and then store them on a local machine. This can be done using any FTP client, including the client built into the Polo program if Polo has already been installed. Once Polo is successfully installed on the local machine, images of a given run can be imported as either an uncompressed directory or a RAR archive. It is important that images are imported to Polo as a directory as Polo assumes relationships between images by their parent directory. For example, if a user wanted to view and classify a single image, they would first create a directory containing only that image and then import that directory into Polo. Additionally, image directories may contain metadata files that describe the conditions of the crystallization experiment. Currently, Polo uses the presence of specific metadata files and file naming schema to differentiate HWI from non-HWI image imports. In the future we hope to increase the number metadata file formats Polo is capable of recognizing and parsing. Lack of this HWI-specific metadata will not prevent import or use of the MARCO model but will limit some of Polo’s more advanced functionality.

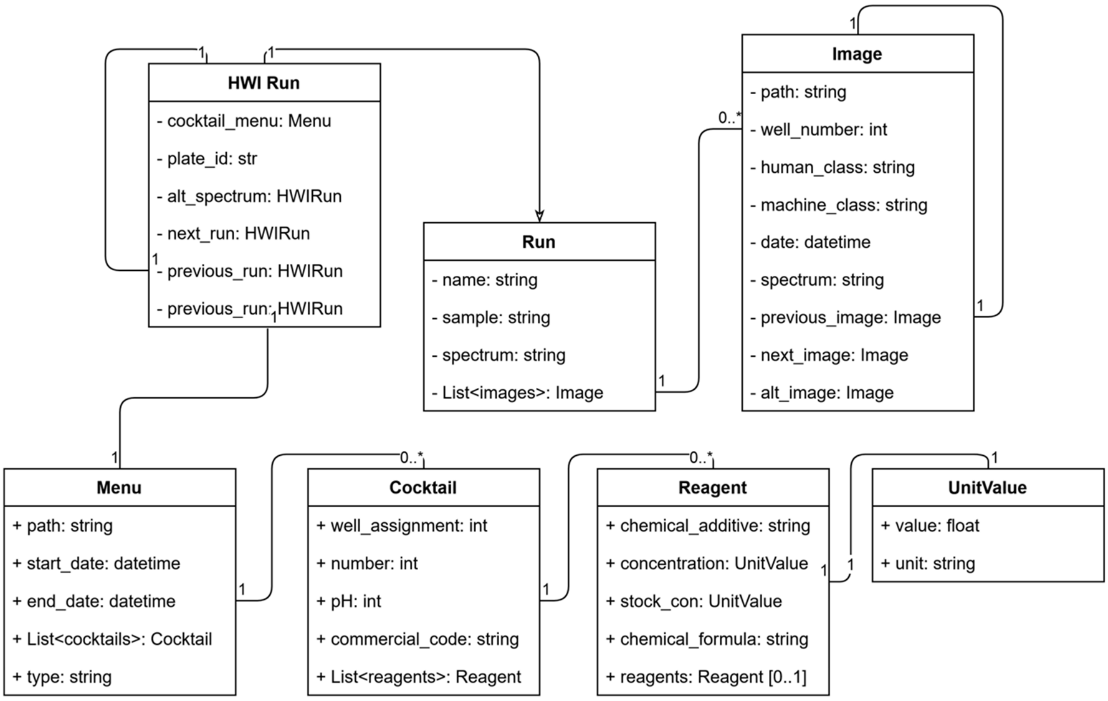

Figure S4 UML diagram of data organizing classes within Polo. For simplicity, classes representing the graphical interfaces and class methods are not shown. Data read from image directories are represented as *Run* objects and subclassed to *HWIRun* objects if the HWI specific metadata files and file naming conventions are present. This metadata provides Polo the information to associate *Image* instances to *Cocktail* instances through the *well_number* attribute which otherwise will hold a null value. Since it is not possible to anticipate all possible crystallization metadata schemas Polo encourages inheritance from *Run* and *HWIRun* objects. Inheriting from the *Run* class should be done when the child class will not have associated cocktail data and *HWIRun* should be inherited from otherwise. As a user interacts with the program and labels images manually or using the MARCO model, these classifications are stored as attributes of *Image* objects. Complete API style code documentation is available at the Polo website.
